# Cross-Species Identification and Validation of Hub Genes and Potential Therapeutic Targets in Myocardial Infarction

**DOI:** 10.3390/ijms27136050

**Published:** 2026-07-06

**Authors:** Zhiyong Sheng, Qiang Li, Mingyu Bao, Wenjing Wang, Zitong Chen, Jiali Guo

**Affiliations:** 1 School of Life Science, Bengbu Medical University, Bengbu 233030, China; 2 Anhui Province Key Laboratory of Cancer Translational Medicine, Bengbu Medical University, Bengbu 233030, China; 3 School of Medical Information and Engineering, Bengbu Medical University, Bengbu 233030, China

**Keywords:** myocardial infarction, hub genes, cross-species analysis, ROC, diagnostic model, biomarkers, therapeutic targets

## Abstract

Myocardial infarction (MI) is a leading cause of mortality worldwide. Identification of robust and translatable molecular markers remains challenging due to inter-dataset and inter-species variability. In this study, we performed a cross-species integrative analysis to identify conserved hub genes and potential therapeutic targets in MI. Analysis revealed five hub genes (IL6, SERPINE1, MMP14, PLAUR, and ENO1), which were consistently validated across human peripheral blood and multiple animal models. A multigene diagnostic model demonstrated strong predictive performance (AUC = 0.904). The model was developed to distinguish myocardial infarction (MI) samples from non-MI control samples in an independent peripheral blood dataset. Drug–gene interaction analysis identified candidate therapeutic compounds. These results are computational predictions from the DGIdb database and do not represent validated therapeutic effects in myocardial infarction. These findings highlight conserved molecular mechanisms of MI and provide potential biomarkers and therapeutic targets with translational relevance.

## 1. Introduction

Myocardial infarction (MI) is a major cardiovascular disorder and continues to be one of the leading global causes of morbidity and mortality [1,2,3,4]. It is primarily caused by the obstruction of coronary blood flow, resulting in ischemic injury and subsequent cardiomyocyte death. The pathological progression of MI involves a series of complex biological processes, involving oxidative stress, inflammatory responses, and extracellular matrix remodeling [5,6,7], and activation of coagulation pathways. Despite advances in early diagnosis and therapeutic strategies, the long-term prognosis of patients with myocardial infarction remains unfavorable, largely due to incomplete understanding of the underlying molecular mechanisms.

In recent years, high-throughput transcriptomic technologies have been widely applied to investigate gene expression changes associated with MI. Numerous studies have identified differentially expressed genes (DEGs) that are potentially involved in MI-related pathological processes. However, findings derived from individual datasets are often limited by sample heterogeneity, technical variability, and species-specific differences. As a result, reproducibility and translational relevance remain major challenges in MI research. Animal models, particularly murine models, are extensively used to study MI mechanisms due to their experimental accessibility and genetic manipulability [8,9]. Nevertheless, significant discrepancies exist between animal models and human conditions, which may limit the clinical applicability of findings. Therefore, identifying genes that are conserved across species is essential for improving the robustness and translational value of molecular signatures in MI. Recent advances allow integration of heterogeneous biological models to identify conserved signals. However, differences between in vitro organoids and in vivo animal models should be considered when interpreting results.

In addition to differential expression analysis, network-based approaches have emerged as powerful tools for identifying key regulatory genes. Protein–protein interaction (PPI) networks provide insights into the functional relationships among genes and allow for the identification of highly connected “hub” genes, which are often critical in disease progression. Furthermore, integrating multiple topological algorithms can enhance the reliability of hub gene identification [10,11].

Another important aspect of translational research is the identification of potential therapeutic targets. Drug–gene interaction databases enable systematic prediction of candidate drugs targeting disease-associated genes [12], thereby facilitating drug repurposing and accelerating therapeutic development. In addition to therapeutic target identification, the development of reliable diagnostic models is another important aspect of translational research. Although several biomarkers have been proposed for myocardial infarction, their diagnostic performance is often limited when used individually. Therefore, integrating multiple gene expression features into predictive models may improve diagnostic accuracy and provide complementary information for clinical assessment.

In this study, we performed a comprehensive cross-species integrative analysis to identify conserved hub genes associated with MI. We first analyzed two human cardiac organoid datasets and two murine MI datasets to identify DEGs within each species. Through homologous gene mapping and intersection analysis, we obtained conserved genes across species. Functional enrichment analysis was conducted to explore their biological roles. Subsequently, a PPI network was established, followed by identification of hub genes using multiple network topology algorithms. The expression levels of these hub genes were examined in the original datasets and further validated across independent datasets and multiple species, including human peripheral blood, engineered heart tissue models, and various animal models. Finally, drug–gene interaction analysis was carried out to explore potential drug candidates associated with the identified hub genes.

Overall, this study aimed to identify robust and conserved molecular signatures of MI and to provide potential targets for therapeutic intervention, thereby improving the understanding of MI pathogenesis and its translational application.

## 2. Results

### 2.1. Identification of Differentially Expressed Genes in Human and Murine MI Models

**Figure 1:**
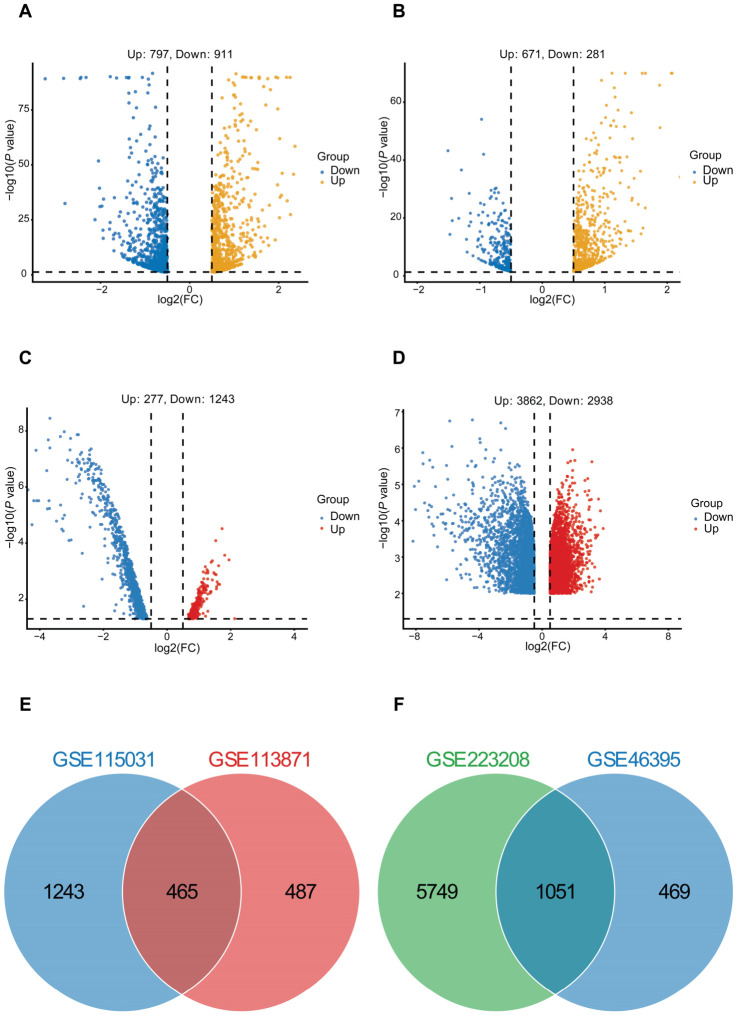
Identification of differentially expressed genes (DEGs) in myocardial infarction models across species. (**A**–**D**) Volcano plots showing DEGs in four GEO datasets, including human cardiac organoid models (GSE115031 and GSE113871) and mouse myocardial infarction models (GSE46395 and GSE223208). The numbers of upregulated and downregulated genes in each dataset are indicated. The *x*-axis represents log2 fold change (log2FC), and the *y*-axis represents −log10 (*p* value). Dashed lines indicate the thresholds for differential expression. (**E**) Venn diagram showing the overlap of DEGs between the two human cardiac organoid datasets (GSE115031 and GSE113871), identifying 465 shared DEGs. (**F**) Venn diagram showing the overlap of DEGs between the two mouse myocardial infarction datasets (GSE223208 and GSE46395), identifying 1051 shared DEGs. DEGs were defined as the union of upregulated and downregulated genes identified in each dataset (|log2FC| ≥ 0.5, adjusted *p* value ≤ 0.05). Shared DEGs represent the intersection of DEGs within each species and were subsequently used for cross-species homolog mapping and downstream integrative analyses.

To identify genes associated with myocardial infarction (MI), differential expression analyses were performed on two human cardiac organoid datasets (GSE115031 and GSE113871) and two murine MI datasets (GSE46395 and GSE223208). Using the criteria of adjusted *p* value ≤ 0.05 and |log2FC| ≥ 0.5, a substantial number of differentially expressed genes (DEGs) were identified in each dataset. Volcano plots demonstrated the distribution of upregulated and downregulated genes in all four datasets (Figure 1A–D).

For cross-dataset integration, DEGs were considered as a combined gene set including both upregulated and downregulated genes. Intersection analysis was then performed independently within each species to reduce dataset-specific bias. A total of 465 shared DEGs were identified between the two human datasets, while 1051 shared DEGs were identified between the two murine datasets (Figure 1E,F). These shared DEGs were subsequently used for cross-species homolog mapping and downstream integrative analyses.

### 2.2. Identification of Conserved Genes Through Cross-Species Integration

**Figure 2:**
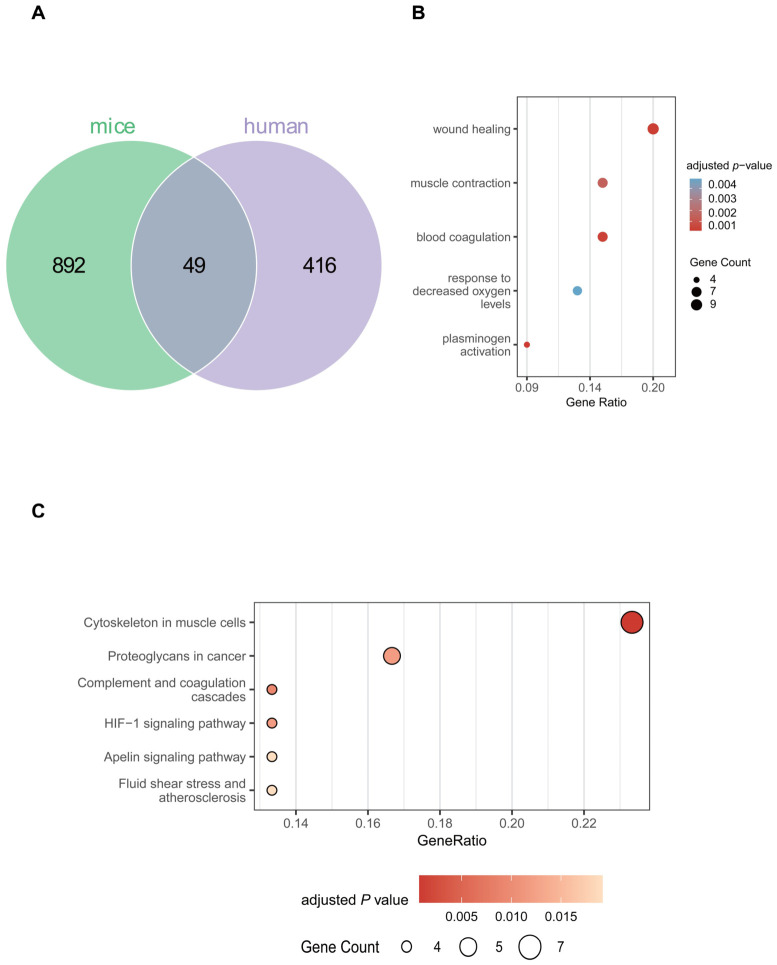
Cross-species integration and functional enrichment analysis of shared genes. (**A**) Venn diagram showing the overlap between human and mouse datasets. Mouse DEGs obtained from the intersection of two myocardial infarction models were first converted to their human homologs and subsequently intersected with the shared DEGs from two human cardiac organoid datasets, yielding 49 common genes. (**B**) Gene Ontology (GO) biological process enrichment analysis of the 49 shared genes. The top enriched terms include wound healing, muscle contraction, blood coagulation, response to decreased oxygen levels, and plasminogen activation. The *x*-axis represents gene ratio, and dot size indicates gene count, while color represents adjusted *p* value. (**C**) Kyoto Encyclopedia of Genes and Genomes (KEGG) pathway enrichment analysis of the 49 shared genes. Significantly enriched pathways include cytoskeleton in muscle cells, proteoglycans in cancer, complement and coagulation cascades, HIF-1 signaling pathway, apelin signaling pathway, and fluid shear stress and atherosclerosis. Dot size indicates gene count, and color represents adjusted *p* value.

To identify conserved molecular signatures across species, the shared murine DEGs were converted to their human homologs and subsequently intersected with the shared human DEGs. This cross-species integration resulted in 49 conserved genes (Supplementary Table S1), which were considered as candidate genes potentially involved in MI across species (Figure 2A). To further explore their biological functions, GO-based functional enrichment analysis demonstrated that these genes were significantly enriched in biological processes including wound healing, blood coagulation, muscle contraction, and response to decreased oxygen levels. Functional enrichment analysis based on KEGG pathways demonstrated that these genes were mainly involved in the HIF-1 signaling pathway, complement and coagulation cascades, apelin signaling pathway, and cytoskeleton-related pathways (Figure 2B,C).

### 2.3. Construction of PPI Network and Identification of Key Interacting Genes

**Figure 3:**
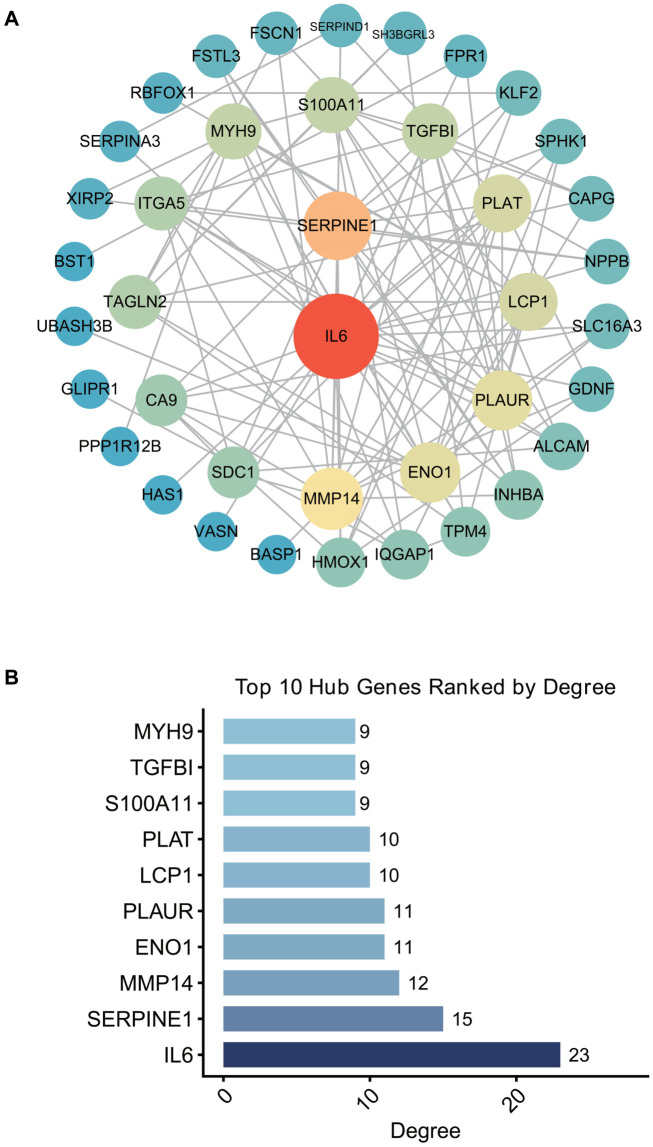
Protein–protein interaction (PPI) network construction and hub gene identification. (**A**) PPI network of the shared genes. Among the 49 cross-species common genes, two housekeeping genes (ACTB and ACTG1) were excluded, and the remaining genes were subjected to PPI network construction using the STRING database. Nodes represent proteins, and edges represent protein–protein interactions. Node size reflects the degree of connectivity, with highly connected genes shown as larger nodes. Node color indicates connectivity or ranking based on network topology analysis. (**B**) Top 10 hub genes ranked by degree centrality in the PPI network. The degree values indicate the number of interactions for each gene, with IL6, SERPINE1, and MMP14 among the most highly connected genes.

To investigate the interactions among conserved genes, a protein–protein interaction (PPI) network was constructed using the STRING database. After excluding housekeeping genes (ACTB and ACTG1), the remaining genes were used to construct the network. A total of 40 genes were included in the PPI network, indicating that not all conserved genes had known interactions (Figure 3A). Network topological analysis based on degree centrality demonstrated that several genes exhibited high connectivity within the network. The top 10 genes ranked by degree are shown in Figure 3B, suggesting that these genes may play important roles in the interaction network.

### 2.4. Identification of Hub Genes Using Multiple Topological Algorithms

**Figure 4:**
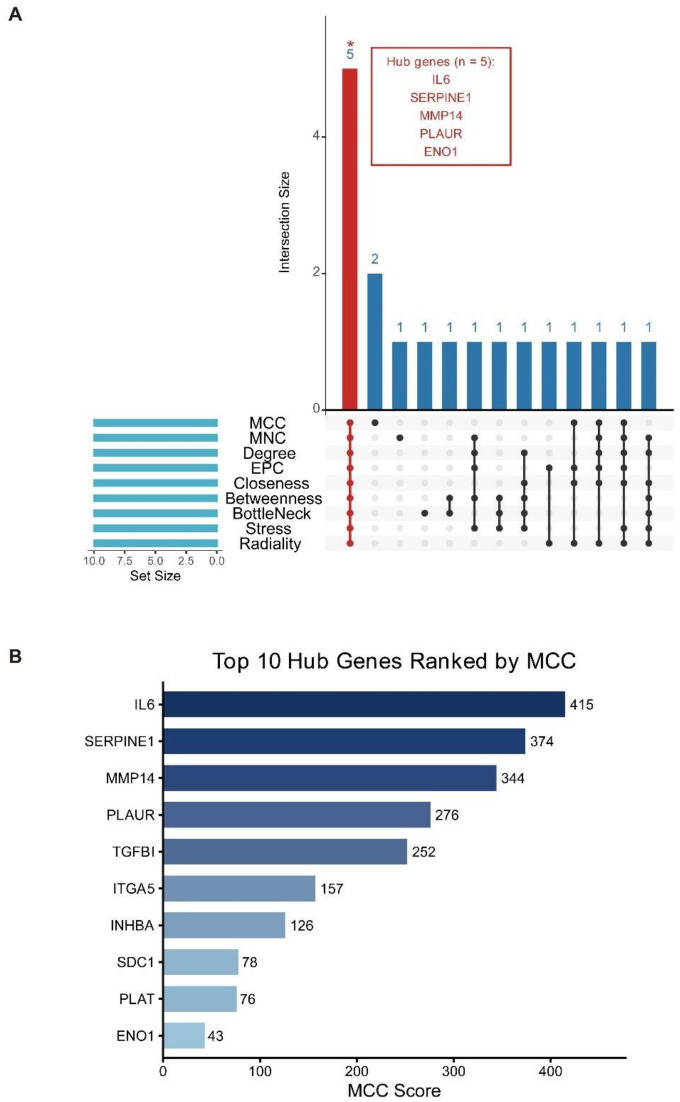
Identification of hub genes using multiple topological algorithms. (**A**) UpSet plot showing the intersection of hub genes identified from the PPI network using nine topological analysis methods in the cytoHubba plugin of Cytoscape, including MCC, MNC, Degree, EPC, Closeness, Betweenness, BottleNeck, Stress, and Radiality. The top 10 genes from each algorithm were selected, and five overlapping genes (IL6, SERPINE1, MMP14, PLAUR, and ENO1) were identified as final hub genes. Asterisks indicate intersecting genes shared across multiple topological methods and do not represent statistical significance. Red bars highlight the final intersection gene set. (**B**) Ranking of the top 10 hub genes based on maximal clique centrality (MCC) scores. IL6, SERPINE1, and MMP14 were among the highest-ranked genes, indicating their central roles in the network.

To further identify key regulatory genes, nine cytoHubba-based topological measures were applied to the PPI network, from which the top 10 genes per algorithm were extracted. The overlap of these gene sets was analyzed using an UpSet plot (Figure 4A). A total of five genes (IL6, SERPINE1, MMP14, PLAUR, and ENO1) were consistently identified across multiple algorithms and were defined as hub genes. Among these, IL6, SERPINE1, and MMP14 exhibited particularly high scores in the maximal clique centrality (MCC) analysis, indicating their central roles within the network (Figure 4B).

### 2.5. Expression Patterns of Hub Genes in MI Datasets

**Figure 5:**
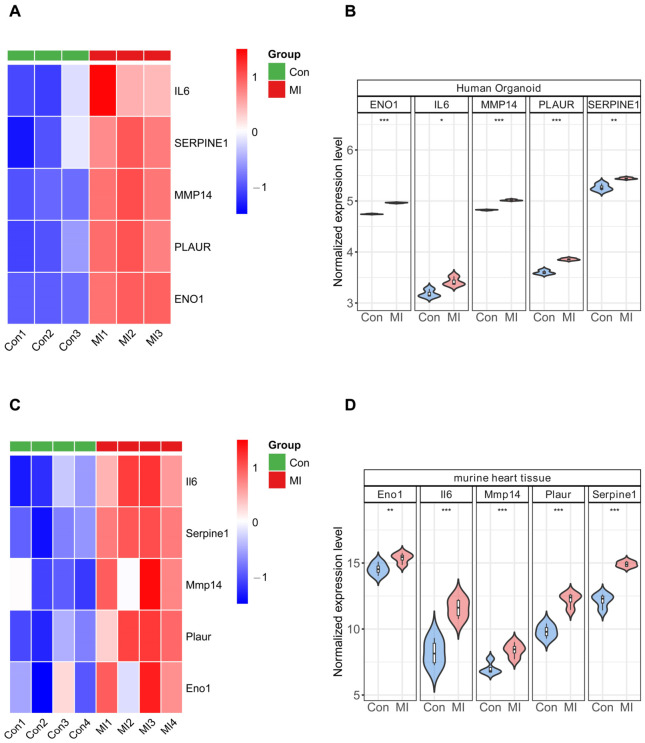
Expression patterns of hub genes in human and murine myocardial infarction models. (**A**) Heatmap showing the expression patterns of the five hub genes (IL6, SERPINE1, MMP14, PLAUR, and ENO1) in human cardiac organoid samples from dataset GSE115031 under control and myocardial infarction (MI) conditions. (**B**) Violin plots illustrating the normalized expression levels of the five hub genes in human cardiac organoid samples (GSE115031), comparing control and MI groups. (**C**) Heatmap showing the expression patterns of the five hub genes in murine heart tissue samples from dataset GSE46395 under control and MI conditions. (**D**) Violin plots illustrating the normalized expression levels of the five hub genes in murine heart tissue samples (GSE46395), comparing control and MI groups. Statistical significance is indicated as * *p* < 0.05, ** *p* < 0.01, *** *p* < 0.001.

The expression of the identified hub genes was examined in the original discovery datasets, including the human cardiac organoid dataset GSE115031 and the murine dataset GSE46395. It should be noted that this analysis was performed within the discovery datasets and therefore represents expression verification rather than independent validation. True external validation was performed in independent datasets across different tissues and species. Heatmap analysis showed distinct expression patterns of the five hub genes between control and MI groups in both human and murine datasets (Figure 5A,C). Violin plot analysis further demonstrated that IL6, SERPINE1, MMP14, PLAUR, and ENO1 were significantly dysregulated in MI samples compared with controls, with consistent trends observed across both species (Figure 5B,D). These results represent myocardial tissue-derived transcriptional alterations reflecting the disease context at the site of injury.

### 2.6. Independent Validation of Hub Genes Across Datasets and Species

**Figure 6:**
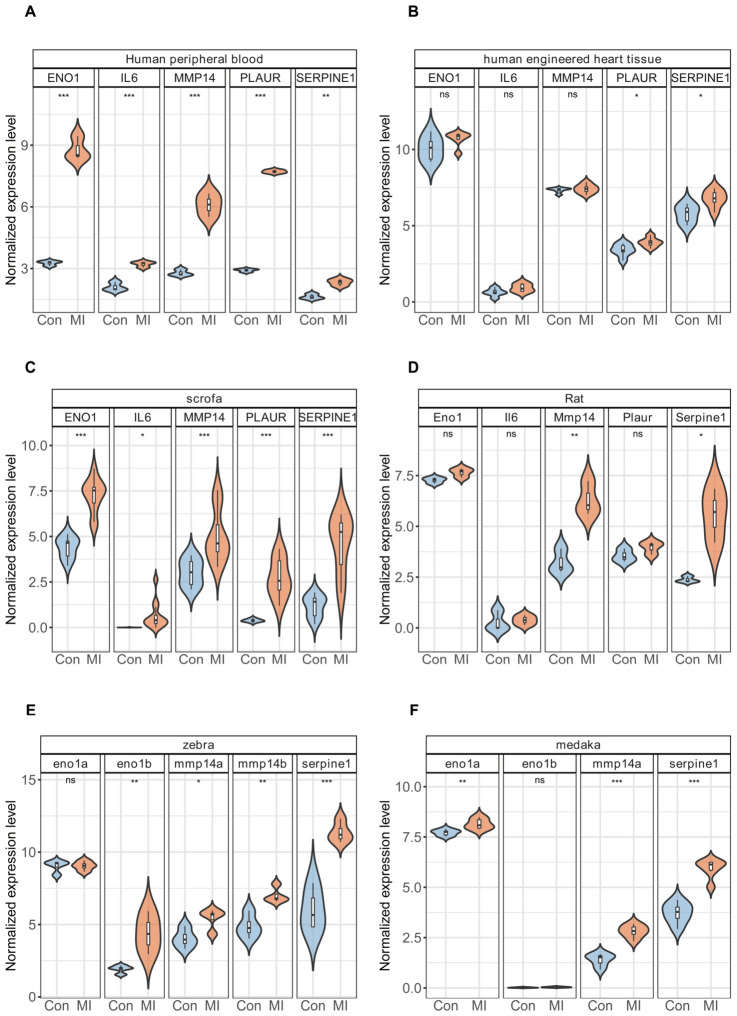
Independent validation of hub genes across multiple datasets and species. (**A**) Violin plots showing the normalized expression levels of the five hub genes (IL6, SERPINE1, MMP14, PLAUR, and ENO1) in human peripheral blood samples under control and myocardial infarction (MI) conditions. (**B**) Expression levels of the five hub genes in human engineered heart tissue models comparing control and MI groups. (**C**) Expression patterns of the five hub genes in porcine (scrofa) myocardial infarction models. (**D**) Expression patterns of the five hub genes in rat myocardial infarction models. (**E**) Expression patterns of homologous genes in zebrafish myocardial infarction models. (**F**) Expression patterns of homologous genes in medaka myocardial infarction models. Statistical significance is indicated as * *p* < 0.05, ** *p* < 0.01, *** *p* < 0.001; ns, not significant.

To evaluate the robustness of the identified hub genes, validation analyses were performed using independent datasets and multiple species. In the human peripheral blood dataset (GSE97320), all five hub genes showed significant differential expression (Figure 6A). In the human engineered heart tissue dataset (GSE234715), most hub genes exhibited consistent expression trends, although some genes showed no significant differences (Figure 6B). In the porcine MI dataset (GSE115665), all five hub genes were significantly dysregulated, further supporting their conservation across species (Figure 6C). In the rat MI dataset (GSE229147), three of the five hub genes showed significant changes, while the remaining genes failed to reach statistical significance (Figure 6D). In zebrafish and medaka models (GSE94617), homologous genes corresponding to hub genes were analyzed. Most genes demonstrated consistent expression patterns; however, certain genes were not included due to the absence of homologous counterparts in these species (Figure 6E,F). Overall, these results indicate that the identified hub genes exhibit cross-species consistency, although species-specific differences were observed. In peripheral blood datasets, gene expression changes should be interpreted as systemic reflections of myocardial injury rather than direct cardiac tissue-specific signals.

### 2.7. Prediction of Potential Therapeutic Drugs Targeting Hub Genes

**Figure 7:**
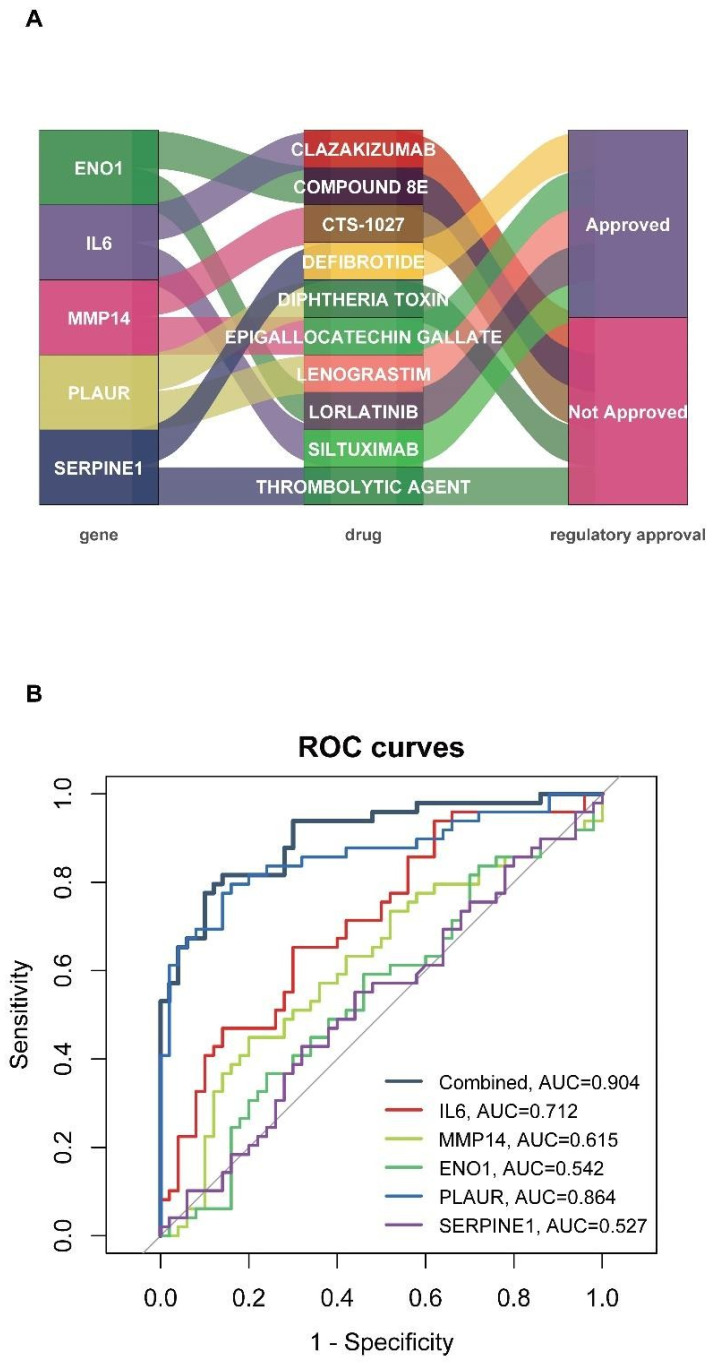
Integrated analysis of diagnostic performance and potential therapeutic targets of hub genes. (**A**) Drug–gene interaction network showing potential therapeutic compounds targeting the five hub genes (IL6, SERPINE1, MMP14, PLAUR, and ENO1), based on the DGIdb database. Nodes represent genes and drugs, and edges indicate known or predicted interactions. Drug approval status is indicated in the figure. (**B**) Receiver operating characteristic (ROC) curves of individual hub genes and the combined model in an independent peripheral blood dataset. The combined model demonstrated superior diagnostic performance (AUC = 0.904) compared with individual genes. The grey diagonal line represents the reference line (AUC = 0.5), and different colors indicate individual genes and the combined model.

To explore potential therapeutic implications, the DGIdb database was used to perform drug–gene interaction analysis. Multiple candidate drugs targeting the identified hub genes were predicted, including both approved and investigational compounds. Representative drugs included siltuximab, clazakizumab, lorlatinib, defibrotide, and epigallocatechin gallate (Figure 7A). The interaction network demonstrated that several hub genes were associated with multiple drug candidates, suggesting their potential as therapeutic targets. These results should be interpreted as computationally predicted drug–gene associations rather than evidence of therapeutic efficacy in myocardial infarction. The findings are intended for hypothesis generation only and should not be interpreted as clinical treatment recommendations.

### 2.8. Diagnostic Performance of the Multi-Gene Model

The diagnostic utility of the identified hub genes was further assessed by constructing a logistic regression model using their expression profiles. The model was applied to an independent peripheral blood dataset (GSE66360) to assess its predictive performance. The combined model demonstrated strong diagnostic accuracy in ROC analysis, yielding an AUC of 0.904 (95% CI: 0.846–0.963) (Figure 7B). Among individual genes, PLAUR exhibited the highest diagnostic accuracy (AUC = 0.864), followed by IL6 (AUC = 0.712), while MMP14, ENO1, and SERPINE1 showed relatively lower predictive performance. The logistic regression coefficients were derived in the independent dataset, where the risk-score model was constructed based on the selected hub genes.

These results indicate that although individual genes have limited diagnostic ability, their combination significantly improves predictive accuracy. However, this model reflects case–control discrimination rather than differential diagnostic or clinical diagnostic performance.

## 3. Discussion

Myocardial infarction (MI) is a complex pathological process involving multiple biological pathways, including inflammation, hypoxia response, coagulation, and extracellular matrix remodeling. In the present study, we performed a comprehensive cross-species integrative analysis to identify conserved molecular signatures associated with MI. By integrating human cardiac organoid datasets and murine MI models, followed by network-based analysis and multi-level validation, we identified five hub genes (IL6, SERPINE1, MMP14, PLAUR, and ENO1) that may play critical roles in MI pathogenesis. It should be noted that human cardiac organoid datasets and murine in vivo MI models differ substantially in biological complexity. Therefore, the integrative analysis in this study does not assume full physiological equivalence between these systems. Instead, it aims to identify conserved transcriptional responses that are robust across heterogeneous experimental contexts, rather than directly modeling identical pathological environments.

A key strength of this study lies in the use of a cross-species integration strategy, which enhances the robustness and translational relevance of the findings. Previous studies have often relied on single datasets or single-species analyses, which are susceptible to variability and limited reproducibility. By intersecting DEGs within species and subsequently integrating homologous genes across species, we identified a set of conserved genes that are more likely to represent fundamental mechanisms of MI. Furthermore, the consistency of these genes across multiple independent datasets and species further supports their biological significance.

Functional enrichment analysis suggested that the conserved genes were strongly implicated in biological processes related to wound healing, blood coagulation, muscle contraction, and response to hypoxia. These results are consistent with the underlying pathophysiology of MI, where hypoxia-related signaling processes, including the HIF-1 pathway, are critically involved in cellular adaptation to ischemic conditions. Similarly, activation of coagulation pathways and extracellular matrix remodeling are key events during infarct healing and cardiac remodeling.

Among the identified hub genes, IL6 is a central pro-inflammatory cytokine that contributes significantly to the inflammatory processes associated with myocardial infarction [13,14,15]. It should be noted that protein–protein interaction networks constructed using STRING may be influenced by literature and database bias. Highly studied genes such as IL6 may appear as highly connected nodes due to extensive prior annotations and therefore should be interpreted in terms of network connectivity rather than causal importance in myocardial infarction. Elevated IL6 expression is closely associated with pathological cardiac remodeling and adverse clinical outcomes. SERPINE1 (PAI-1) is a central mediator of fibrinolysis and is strongly implicated in thrombosis and vascular remodeling [16,17]. Increased expression of SERPINE1 may contribute to impaired fibrinolysis and increased risk of thrombosis after MI.

MMP14 and PLAUR contribute to extracellular matrix remodeling through regulation of matrix degradation and tissue reorganization. Matrix metalloproteinases (MMPs) are known to regulate cardiac remodeling by modulating extracellular matrix turnover [18], while PLAUR plays a role in plasminogen activation and cell migration [19]. Dysregulation of these genes may contribute to pathological remodeling and scar formation following MI.

In addition, ENO1, a glycolytic enzyme, has been reported to participate in metabolic reprogramming and stress responses under hypoxic conditions [20,21]. Its upregulation in MI may reflect adaptive metabolic changes in response to ischemic stress. The convergence of these five genes may reflect coordinated inflammatory activation and extracellular matrix remodeling processes during myocardial infarction, driven by shared regulatory programs and immune–stromal interactions. The hub gene signature mainly reflects systemic inflammatory and multicellular responses rather than cardiomyocyte-specific signals. In addition, bulk RNA-seq analysis can not distinguish cell-type-specific expression changes, and the observed upregulation of genes such as IL6 and PLAUR may be partially attributed to altered immune cell composition, including neutrophil and macrophage infiltration.

Importantly, the expression patterns of these hub genes were not only observed in the discovery datasets but were also validated across multiple independent datasets and species [22,23], including human peripheral blood, engineered heart tissue models, and various animal models. This multi-level validation significantly strengthens the reliability of our findings. Notably, although most genes showed consistent expression trends across species, some variability was observed, which may be attributed to species-specific differences or the absence of homologous genes in certain models.

Another important aspect of this study is the exploration of potential therapeutic targets. Drug–gene interaction analysis highlighted several potential therapeutic agents targeting the hub genes, including both approved and investigational compounds [24]. For example, siltuximab and clazakizumab, which target IL6 signaling, have been investigated in inflammatory diseases and may have potential applications in MI. Similarly, other compounds identified in this study may provide opportunities for drug repurposing. These drug–gene interactions are in silico predictions and should be interpreted as hypothesis-generating associations rather than confirmed therapeutic targets. As above, these findings should be considered hypothesis-generating and require further experimental validation before any therapeutic conclusions can be drawn. In addition to the identification of hub genes, we constructed a multi-gene diagnostic model to evaluate their combined predictive value. The model demonstrated strong diagnostic performance in an independent peripheral blood dataset (AUC = 0.904). This model was designed to discriminate myocardial infarction (MI) samples from non-MI control samples based on transcriptomic profiles, demonstrating good discriminative ability. It should be noted that the datasets used in this study are cross-sectional, and detailed post-MI time-course information was not available. The modeling strategy was based on feature selection from discovery datasets followed by model construction in an independent cohort, which may reduce dataset-specific bias. Notably, while individual genes showed variable and sometimes limited performance, their integration significantly improved prediction accuracy. This finding highlights the importance of multi-gene signatures in complex diseases such as myocardial infarction.

However, it should be noted that the model was developed based on transcriptomic data, and further validation using clinically applicable platforms, such as RT-qPCR, as well as larger patient cohorts, is required before potential clinical application.

Despite these strengths, several limitations should be acknowledged. First, this study is primarily based on publicly available transcriptomic datasets, and experimental validation is lacking. Second, although cross-species integration improves robustness, differences in experimental conditions, sample sources, and species-specific biology may still influence the results. Third, the functional roles of the identified hub genes in MI require further investigation through in vitro and in vivo experiments. Overall, this study represents a hypothesis-generating and exploratory bioinformatics analysis based on publicly available transcriptomic datasets.

## 4. Materials and Methods

### 4.1. Data Collection and Preprocessing

Gene expression data for myocardial infarction (MI) were retrieved from the GEO repository (National Center for Biotechnology Information, Bethesda, MD, USA; https://www.ncbi.nlm.nih.gov/geo/, accessed on 5 July 2026). Two human cardiac organoid datasets (GSE115031 and GSE113871) and two murine MI datasets (GSE46395 and GSE223208) were included for discovery analysis. For validation, additional datasets derived from human peripheral blood (GSE97320), engineered heart tissue models (GSE234715), and multiple species, including porcine (GSE115665), rat (GSE229147), and zebrafish/medaka (GSE94617), were collected from the GEO database. In addition, the human peripheral blood dataset (GSE66360) was used as an independent validation cohort for diagnostic model evaluation. R software (version 4.5.1) was used for all analyses. Raw expression matrices were normalized according to platform-specific standards. Probe IDs were mapped to gene symbols based on annotation files, and average expression values were calculated for genes represented by multiple probes. Probes without corresponding gene symbols were removed.

### 4.2. Identification of Differentially Expressed Genes (DEGs)

For each dataset, differential expression analysis was independently carried out using the limma package in R [25]. Genes satisfying the criteria of adjusted *p* value (adjust *p*) ≤ 0.05 and absolute log2 fold change (|log2FC|) ≥ 0.5 were defined as differentially expressed genes (DEGs). This threshold was chosen to balance biological effect size and gene retention for cross-species integrative analysis. A more stringent cutoff (|log2FC| ≥ 1.0) markedly reduced the number of shared DEGs across datasets, limiting subsequent cross-species integration, PPI network construction, and hub gene identification. Volcano plots were generated to visualize the distribution of DEGs in each dataset. To improve robustness and reduce dataset-specific bias, DEGs identified from the two human datasets (GSE115031 and GSE113871) were intersected to obtain shared human DEGs. Similarly, DEGs from the two murine datasets (GSE46395 and GSE223208) were intersected to obtain shared murine DEGs. To further enhance robustness, genes consistently observed across multiple datasets and species were retained for downstream analysis. For DEG integration, upregulated and downregulated genes were not analyzed separately. Instead, DEGs from each dataset were combined as a single gene set, and intersection analysis was performed within each species to identify shared DEGs prior to cross-species homolog mapping.

### 4.3. Cross-Species Homologous Gene Mapping

To identify conserved genes across species, shared murine DEGs were translated into their human homologs via the biomaRt package in R. Gene annotation and ortholog mapping were performed using Ensembl (December 2021 archive, https://dec2021.archive.ensembl.org/ accessed on 5 July 2026). The homolog-converted murine genes were then intersected with shared human DEGs to obtain cross-species conserved genes for downstream analyses [26]. To minimize annotation bias, only one-to-one orthologs with high-confidence mappings were retained, and genes with ambiguous or multiple homolog assignments were excluded.

### 4.4. Functional Enrichment Analysis

The clusterProfiler package in R was used to perform functional enrichment analysis. GO biological process and KEGG pathway enrichment were applied to characterize the biological functions of conserved genes [27,28]. An adjusted *p* value threshold of <0.05 was used to determine statistical significance. Bubble plots were generated to visualize gene ratio, gene number, and adjusted *p* values.

### 4.5. Construction of Protein–Protein Interaction (PPI) Network

The STRING database (https://string-db.org/) was used to construct a protein–protein interaction (PPI) network [10]. To avoid bias caused by highly connected housekeeping genes, ACTB and ACTG1 were excluded prior to network construction. The interaction network was constructed using a minimum confidence score of 0.4 and subsequently imported into Cytoscape (version 3.9.1) for visualization and downstream analysis [29].

### 4.6. Identification of Hub Genes Using CytoHubba

The cytoHubba plugin in Cytoscape was used to identify hub genes. Network topology was evaluated using nine algorithms, namely, MCC, MNC, Degree, EPC, Closeness, Betweenness, BottleNeck, Stress, and Radiality [11]. For each algorithm, the top 10 ranked genes were selected. The intersection of these gene sets was visualized using an UpSet plot. Genes consistently identified across multiple algorithms were defined as hub genes.

### 4.7. Expression Analysis of Hub Genes

The expression patterns of hub genes were examined in the original discovery datasets, including human cardiac organoid dataset GSE115031 and murine dataset GSE46395. Heatmaps and violin plots were generated using the pheatmap and ggplot2 packages in R to visualize differences between the control and MI groups.

### 4.8. Independent Validation Across Datasets and Species

To evaluate the robustness and cross-species conservation of hub genes, validation analyses were performed in independent datasets, including human peripheral blood, human engineered heart tissue models, porcine MI models, rat MI models, zebrafish MI models, and medaka MI models. For non-human species, homologous genes were used when direct gene matches were unavailable. Student’s t-test or the Wilcoxon rank-sum test was applied to assess statistical differences between the control and MI groups, depending on the distribution of the data.

### 4.9. Drug–Gene Interaction Analysis

Drug–gene interactions were predicted using the Drug–Gene Interaction Database (DGIdb) (https://dgidb.org/) [30]. The identified hub genes were used as input, and associated drugs were retrieved. Both approved and investigational drugs were included. The interaction network was visualized, and drug approval status was annotated.

### 4.10. Diagnostic Model Construction and Evaluation

To evaluate the diagnostic performance of the five hub genes (IL6, MMP14, ENO1, PLAUR, and SERPINE1), a logistic regression model was established. The risk score was defined as follows: Risk score = −22.226 + 1.078 × IL6 + 0.069 × MMP14 + 0.248 × ENO1 + 1.312 × PLAUR + 0.777 × SERPINE1. Samples were classified into high- and low-risk groups using the median risk score as the cutoff value. Receiver operating characteristic (ROC) analysis was performed using the pROC package in R to evaluate the diagnostic performance of the model. The area under the curve (AUC) and corresponding 95% confidence intervals (CIs) were calculated in an independent peripheral blood dataset (GSE66360). These analyses reflect case–control classification based on transcriptomic features rather than clinical diagnostic decision-making.

### 4.11. Statistical Analysis

All analyses were carried out in R software (version 4.5.1). Statistical significance was defined as a two-sided *p* value < 0.05 unless otherwise specified.

## 5. Conclusions

In conclusion, this study identified five conserved hub genes associated with myocardial infarction through cross-species integrative analysis and multi-level validation. These genes are involved in key pathological processes, including inflammation, coagulation, and extracellular matrix remodeling. Our findings provide new insights into the molecular mechanisms of MI and highlight potential biomarkers and therapeutic targets for future research and clinical applications.

## Data Availability

The datasets analyzed in this study are publicly available in the Gene Expression Omnibus (GEO) database under accession numbers GSE115031, GSE113871, GSE46395, GSE223208, GSE97320, GSE234715, GSE115665, GSE229147, GSE94617, and GSE66360.

## Supplementary Materials

The following supporting information can be downloaded at https://www.mdpi.com/article/10.3390/ijms27136050/s1.
