# Supplementary material for: Cross-Species Identification and Validation of Hub Genes and Potential Therapeutic Targets in Myocardial Infarction
Source: Int J Mol Sci. 2026 Jul 6;27(13):6050. doi: 10.3390/ijms27136050 (PMC13362447; doi:10.3390/ijms27136050)
Supplement: Supplementary file 1 [file ijms-27-06050-s001.zip › ijms-4356359-supplementary.pdf]

Supplementary Table S1. List of the 49 conserved genes identified by cross-species integration analysis.

Gene Symbol
LCP1
SERPINA3
HMOX1
FIBIN
ITGA5
SERPINE1
FJX1
FSCN1
GDNF
GRIP2
ALCAM
CA9
ENO1
INHBA
BASP1
SERPIND1
ACTB
PLAUR
BST1
KLF2
RAVER2
TGFBI
MYH9
TPM4
MMP14
IL6
UBASH3B
VASN
SPHK1
XIRP2
PLAT
TNFRSF10D
ACTG1
S100A11
HAS1
IQGAP1
TRIM46
SH3BGRL3
FPR1
SLC16A3

SDC1
GLIPR1
FSTL3
NPPB
RBFOX1
PPP1R12B
LMOD3
CAPG
TAGLN2
